# Variability of antioxidant and biological activities of Rhus tripartitum related to phenolic compounds

**DOI:** 10.17179/excli2016-735

**Published:** 2017-03-31

**Authors:** Hanène Ben Miled, Mariem Saada, Ines Jallali, Zaineb Ben Barka, Mounira Tlili, Hichem Alimi, Mohsen Sakly, Khémais Ben Rhouma, Manef Abderrabba, Hafedh Abdelmelek, Olfa Tebourbi, Riadh Ksouri

**Affiliations:** 1 Laboratoire des Plantes Aromatiques et Médicinales (LPAM), Centre de Biotechnologie, Technopôle de Borj Cédria (CBBC), BP 901, 2050, Hammam-Lif, Tunisie; 2 Laboratoire de Physiologie Intégrée, Faculté des Sciences de Bizerte, Université de Carthage, 7021, Jarzouna, Tunisie; 3 Laboratoire Matériaux Molécules et Applications, IPEST, Université de Carthage BP51, 2070 La Marsa, Tunisie

**Keywords:** Rhus tripartitum, phenolics, anticancer ability, antioxidant capacity, anti-inflammatory activity

## Abstract

Rhus species are known in traditional medicine for their therapeutic virtue and their extracts showed numerous important properties including antimalarial, antimicrobial, antiviral, and hypoglycemic and anticonvulsant activities. Rhus tripartitum (Ucria) is a medicinal plant widely used in Tunisia folk medicine against chronic diarrhea and gastric ulcer. This study was designed to examine *in vitro *and* ex vivo* antioxidant, anti-inflammatory and anticancer activities of four extracts of Rhus tripartitum root cortex with increasing solvent polarity (hexane, dichloromethane, methanol and water). HPLC was used to identify and quantify phenolic compounds in Rhus extract. Water extract showed the highest antioxidant activity using oxygen radical absorbance capacity (ORAC method) with 8.95 ± 0.47 µmol Trolox/mg and a cell based-assay with 0.28 ± 0.12 µmol Trolox/mg as compared to the other fractions. Moreover, methanol extract displayed the strongest anti-cancer activity against human lung carcinoma (A-549) and colon adenocarcinoma cell lines (DLD-1) with an IC_50 _value of 60.69 ± 2.58 and 39.83 ± 4.56 µg/ml (resazurin test) and 44.52 ± 5.96 and 55.65 ± 6.00 µg/ml (hoechst test), respectively. Besides, the highest anti-inflammatory activity, inhibiting nitric oxide (NO) release, was exhibited by dichloromethane extract with 31.5 % at 160 µg/ml in lipopolysaccharide (LPS)-stimulated RAW 264.7 macrophages. The HPLC analysis showed that catechol and kaempferol were the major phenolics. These data suggest the richness of all fractions of Ucria root on interesting bioactive molecules with different polarity and confirm the known traditional therapeutics virtues of this species for the treatment of dysentery, diarrhea and gastric ulcer.

## Introduction

The human body is equipped with many antioxidant defense systems to preserve healthy cell membranes against free radicals and active oxygen species (Kaur and Kapoor, 2001[13]). A large diversity of free radical scavenging molecules found in plants, such as phenolic compounds, carotenoids, vitamins, and some other endogenous metabolites, are found to exhibit powerful antioxidant activity (Cai et al., 2003[2]). Therefore, antioxidant compounds provided by the diet may not only retard oxidative degradation of lipids and thereby promote food quality and nutritional value (Ksouri et al., 2009[18]), but also enrich the antioxidative status of living cells and therefore decrease the damage, mainly in the elderly (Shukla et al., 1997[36]). Phenolic compounds as powerful antioxidants in plants play a crucial role to quench reactive oxygen species responsible in oxidative stress, these bioactive antioxidants are increasing and may reach 8000 (Havsteen, 2002[10]; Wollgast and Anklam, 2000[43]). Previous studies have shown that many of these antioxidant compounds possess biological activities such as anti-inflammatory, anti-atherosclerotic, antitumor, antimutagenic activities (Sala et al., 2002[33]). Besides, it has been shown that there might be a relation between the intake of natural antioxidants and the lowering of risks of several diseases related to ageing like cancer, cardiovascular disease, and diabetes (Ksouri et al., 2012[19]; Yang et al., 2001[44]). Accordingly, some phenolic antioxidants are assumed as preventive and curative mediators against UV-radiations (Weiss and Landauer, 2003[42]) which are responsible for skin diseases (Eberhardt et al., 2000[6]; Ganesan et al., 2011[7]). In Tunisia, *Rhus tripartita (*Ucria) Grande is a known medicinal plant used mainly for the treatment of diarrhea and dysentery (Abbassi and Hani, 2012[1]). *Rhus tripartitum* root cortex extract was reported to contain interesting phenolics (proanthocyanidic oligomers and polymers) with strong antioxidant capacity and prevent thymocytes apoptosis in rat (Tebourbi et al., 2006[39]). Other phenolic compounds have been identified in this plant which exhibit anti-inflammatory and antimicrobial properties (Mahjoub et al., 2006[23]; Abbassi and Hani, 2012[1]). So far, no other study of biological activities of *Rhus tripartitum *root was performed, that's why we propose to investigate *in vitro* and *ex vivo* antioxidant, anti-inflammatory and anticancer abilities and to identify and quantify their phenolic content using HPLC system after extraction by soxhlet using different solvents.

## Materials and Methods

### Preparation of crude plant extracts 

Roots of *Rhus tripartita* (Ucria) Grande [=R. tripartitum (Ucria) D.C. = R. oxya-canthoides Dum. Cours. = R. oxyacantha Shousb. Ex. Cav.] (Pottier-Alapetite, 1979[31]; Le Floc'h and Boulous, 2008[20]) were collected in April 2012 from Labayadh-Hajeb layoun city. This medicinal plant was identified at the Biotechnology Centre of Borj-Cédria, and a voucher specimen (RT-CBBC-53) was placed in our Laboratory. To assess biological activities, 30 g of powdered roots were extracted in a soxhlet system with 4 increasing polarity solvents (hexane, dichloromethane, methanol and water). Then, root extracts were filtered, and the solvent was evaporated using rotary vacuum evaporator. At last, root samples were lyophilized and the residue was dissolved in dimethyl sulphoxide prior to analysis.

### Assessment of antioxidant activities

#### ORAC_FL_ assay

The procedure was modified from the method described by Ou et al. (2001[29]). The ORAC assay was accomplished in black round bottom 96-well microplates (Costar) on a Fluoroskan Ascent FL™ plate reader (Labsystems) using an automated injector. The test was directed at 37.5 °C and in pH 7.4 phosphate buffer, with a blank sample in parallel. Different concentrations of Trolox as the control standard were used in quadruplicate, and a gradient of 16 concentrations of the extracts was set without replication. The fluorimeter was planned to register the fluorescence (λ ex.: 485 nm/em.: 530 nm) of fluorescein each minute once adding of 375 mM of 2,2-azobis (2-amidinopropane) dihydrochloride (AA-PH), for 35 min. The final results were calculated using the net area under the curves of the extract concentrations for which reduction of at least 95 % of fluorescence was detected at 35 min and which also showed a linear dose-response pattern. ORAC values were stated in micromoles of Trolox equivalents (TE) per gram (µmol TE/g).

### Antioxidant cell assay using 2', 7'-dichlorofluorescin-diacetate (DCFH-DA)

Antioxidant activity was assessed via the DCFH-DA test as reported by Legault et al. (2003[21]), with changes. Human skin fibroblast cells were plated in 96 microwell plates at 10,000 cells per well and incubated for 48 hrs at 37 °C and 5 % CO_2_. Next, the cells were washed with 150 µl Hank's balanced salt solution (HBSS) at pH 7.4 and incubated for 30 min with 100 µl HBSS (pH 7.4) holding 5 µM DCFH-DA (Sigma-Aldrich). Then, the cells were washed with 150 µl HBSS. To assess antioxidant activity, the cells were incubated either with a growing concentration of samples from *Rhus tripartitum*, quercetin or Trolox, in the presence or absence of 200 µM tert-butylhydroperoxide (tBH). Fluorescence was quantified after 1 and 4 hrs on the automated 96-well plate reader (Fluoroskan Ascent FL™, Labsystems) via an excitation wavelength of 485 nm and an emission wavelength of 530 nm.

### Cell culture

The human lung carcinoma A-549 (ATCC #CCL-185) and colon adenocarcinoma DLD-1 (ATCC #CCL-221) cell lines were purchased from the American Type Culture Collection (ATCC, Manassas, USA). The A-549 and DLD-1 cell lines were developed in Minimum Essential Medium with Earle's salts. The media was complemented with 10 % foetal calf serum (Hyclone, Logan, USA) for (A-549 and DLD-1), solution of vitamins (1×), sodium pyruvate (1×), nonessential amino acids (1×), penicillin (100 IU) and streptomycin (100 µg/ml) (Mediatech Cellgro). Cells were cultivated in a humidified atmosphere at 37 °C in 5 % CO_2_.

### Cytotoxicity assay

Exponentially growing cells were plated at a density of 5×10^3^ cells per well in 96-well microplates (Costar, Corning Inc.) in 100 µl of culture medium and were permitted to paste for 16 hrs before treatment. Then, 100 µl of growing concentrations of sample dissolved in the suitable solvent (DMSO) were added. The final concentration of solvent in the culture medium was preserved at 0.5 % (v/v) to evade solvent toxicity. The cells were incubated for 48 hrs in the presence or in the absence of sample. Cytotoxicity was measured by means of the resazurin reduction test as reported by O'Brien et al. (2000[28]). Fluorescence was quantified on an automated 96-well Fluoroskan Ascent Fl™ plate reader (Labsystems) with an excitation wavelength of 530 nm and an emission wavelength of 590 nm. Cytotoxicity was stated as the concentration of extract inhibiting cell growth by 50 % (IC_50_).

### Measurement of anti-inflammatory activity by nitrite quantification

Exponentially growing cells were plated in 24-well microplates (BD Falcon) at a density of 2 ×10^5^ cells per well in 400 µl of culture medium and were allowed to adhere overnight. Cells were then processed with or without N(G)-nitro-L-arginine methyl ester (L-NAME) as positive control, or growing concentrations of samples dissolved in the proper solvent, and incubated at 37 °C, 5 % CO_2_ for 24 hrs. The ultimate concentration of solvent in the culture medium was sustained at 0.5 % (v/v) to evade solvent toxicity. Then, cells were stimulated with 100 µg/ml lipopolysaccharide (LPS). After 24 hrs, cell-free supernatants were gathered and deposited at 80 °C until NO measurement using the Griess reaction (Green et al., 1990[9]) with slight changes. In the beginning, 100 µl aliquots of cell supernatants were incubated with 50 µl of 1 % sulphanilamide and 50 µl of 0.1 % N-1-naphtylethylenediamine dihydrochloride in 2.5 % H_3_PO_4 _at room temperature for 20 min. Then, absorbance at 540 nm was evaluated by an automated 96-well Varioskan Ascent plate reader (Thermo Electron) and the existence of nitrite was measured by comparison with an NaNO_2_ standard curve.

### High performance liquid chromatography analysis

The identification of phenolic compounds in methanol and water root fractions was done using an HPLC system equipped with a Hypersil ods-C18 analytical column of 4.6 x 100 mm and 0.5 μm particle size. Column temperature was sustained at 25 °C. The flow-rate of mobile phase was 0.7 mL/min and the injected extract volume was 2 μl. Mobile phase C was milli-Q, water was composed of 0.2 % formic acid and mobile phase B was acetonitrile. The gradient program was as follows: 35B/65C (0-6 min); 60B/40C (6-9 min); 80B/20C (9-14 min); 100B/0C (14-25 min) and 35B/65C (25-30 min). The different detected compounds were identified by comparing their retention time with those of injected authentic standards.

### Statistical analysis

Means were compared statistically via the PRISMA PAD program (version 5), with Student's t-test at the P < 0.05 significance level. A one-way analysis of variance (ANOVA) and Newman-Keuls multiple range test were carried out to test any significant difference between species at P < 0.05.

## Results and Discussion

### In vitro and ex vivo antioxidant activity in several extracts of Rhus tripartitum roots

ORAC and DCFH are among the most reliable *in vitro *and* ex vivo* antioxidant test respectively. In fact, the oxygen radical absorbance capacity (ORAC) method has been found to be the most relevant one for biologic samples (Huang et al., 2005[11]). Besides, the rapid cell-based assay using dichlorofluorescin (DCFH) oxidation indirectly measure the effect of intracellular antioxidant activities in scavenging the reactive oxygen species (ROS) and assess the pro- and antioxidant potential of various pure compounds, fruits and vegetable juices (Girard-Lalancette et al., 2009[8]). Results (Table 1(Tab. 1)) showed that root water extract exhibited a potent antioxidant activity that could inhibit the tBH-induced oxidation of DCFH with an IC_50_ value of 0.28 µg/ml. Contrariwise, inhibition of DCFH-oxidation by methanol, hexane and dichloromethane extract was lower (respectively, 14.39, 35.25 and 20.52 µg/ml). In addition, this value of water extract is higher than those found in other plants (Suaeda fruticosa, Zygophyllum album and Solanum elaeagnifolium) from Tunisia (Oueslati et al., 2012[30]; Ksouri et al., 2013[17]; Mejri et al., 2014[25] respectively) suggesting the strongly pro-oxidation effect of Rhus tripartitum roots. The antioxidant activity of root extracts (hexane, dichloromethane, methanol and water) was assessed *in vitro* using the ORAC assay. Results indicate that water and methanol extracts have statistically similar and strong antioxidant activity with respectively an ORAC value of 8.95 and 8.55. Quercetin, used as an antioxidant standard exhibited the highest ORAC value (21.45 µmol Trolox/ml). Hexane and dichloromethane extract exhibited a lower activity with ORAC values of 0.11 and 0.06 µmol Trolox. These results clearly demonstrate the superiority of polar extract (water and methanol) to non-polar ones (hexane and dichloromethane) and that this antioxidant activity may be due to the richness of water extract mainly in phenolic compounds. Previous works on edible plants showed that polar extract has a better ORAC activity than non-polar one (Oueslati et al., 2012[30]; Ksouri et al., 2013[17]). Moreover, a comparison of antioxidant activities of guava fruit extracts showed a superiority of the methanol extract against dichloromethane extract (Thaipong et al., 2006[40]). Accordingly, Kratchanova et al. (2010[16]) reported the important impact of solvent extracting on the assessment of antioxidant activity and phenolic content, suggesting the use of several extraction methods to improve the investigation of the antioxidant activity of the natural compound.

### Evaluation of Rhus tripartitum roots cortex extract cytotoxicity against tumor cell lines 

Over the past years, there was a lot of focus on natural compound as potential drugs to prevent or treat pathologies such as cancer which was always associated with inflammatory responses (Kim et al., 2012[14]) and suggesting that inflammation played a crucial role in cancer progress. Extracts from *Rhus* root cortex were evaluated on the anticancer effects with the purpose of better understanding their relationship with their anti-inflammatory effect. In this context, the methanol, hexane, dichloromethane and water extracts of *Rhus tripartitum* root cortex were subjected to *in vitro* screening, to evaluate their potential cytotoxic activity against human cancer cell lines. The results presented in Table 2(Tab. 2) showed that, in particular, the methanol extract inhibited the proliferation of the tumor cell lines unlike other three extracts which were poorly active against carcinoma cell lines. Also, respectively, the IC_50_ values of methanol extract active against the two carcinoma A-549 and DLD-1(60.69 and 39.83µg/ml), were comparable to those of etoposide used as standard (35.52 µg/ml). Contrariwise, other recent research reported that hexane (Tundis et al., 2011[41]) and dichloromethane (Oueslati et al., 2012[30]; Ksouri et al., 2013[17]) extracts were mostly active against the two carcinoma and showed no significant cytotoxicity towards healthy human skin fibroblast cell lines WS1. In addition, methanolic twig extract from *Ledum groenlandicum* was also active against DLD-1 colon carcinoma and A-549 lung carcinoma cells (Dufour et al., 2007[5]). Altogether, these results advocate the appreciable anti-tumour activity of *Rhus tripartitum* which prompt it to be considered as a potential source of anticancer compounds.

### Evaluation of the anti-inflammatory activity of Rhus tripartitum extracts on LPS-activated RAW 264.7 macrophages

Phenolic compounds have been considered valuable in the treatment of chronic inflammatory illnesses related to over manufacturing of nitric oxide (NO) (Jiang and Dusting, 2003[12]). Lipopolysaccharide (LPS), initiates several signaling paths in macrophages and improves creation of inflammatory mediators (Sun and Stenken, 2003[37]).The induction of iNOS and overgeneration of NO results from the stimulation of RAW 264.7 macrophages by LPS and could be identified and quantified photometrically. In our study, all extracts inhibit partially NO release and dichloromethane fraction (31.5 %) exhibited the most active anti-inflammatory effect among all fractions, followed by hexane extract (28.1 %), methanol (17 %) and Water extract (12.5 %) at a concentration of 160 µg/ mL (Table 3(Tab. 3)). L-NAME (250 µM) used as positive control significantly inhibited NO release (62.5 %). All extracts did not exhibit cytotoxicity towards RAW 264.7 cells even at the highest dose. Many previous studies have shown that dichloromethane extracts or fractions had good anti-inflammatory activity and in occasion better than other polar solvents (Schinella et al., 1998[35]; Mat Ali et al., 1998[24]; Miño et al., 2004[26]). In fact, it was reported that the anti-inflammatory capacity against NO production in LPS-induced RAW 264.7 macrophages may be due to the synergistic or even, the additive effects of chemical constituents in the plant mixture (Yang et al., 2013[45]). On the other hand, it was reported that overproduction of NO might direct to the improved replication of genes and oxidative damage to DNA and consequently take part in the oncogenesis procedure (Liu and Hotchkiss, 1995[22]; Tamir and Tannenbaum, 1996[38]). In this work, methanol extract not only restrained significantly NO production but also effectively exerted cytotoxicity against DLD-1 colon carcinoma and A-549 lung carcinoma cells which could be due, in part, to the presence of phenolic compounds. The results indicated that there might be some relationship between inhibitory activities on NO production and cytotoxic properties towards cancer cell lines, which was in disagreement with the deduction of Kim et al. (2012[14]). Additional investigations will be needed to establish the specific mechanism of the extract on cytotoxic activities against cancer cell lines and anti-inflammatory activities.

### HPLC analysis

**Figure 1:**
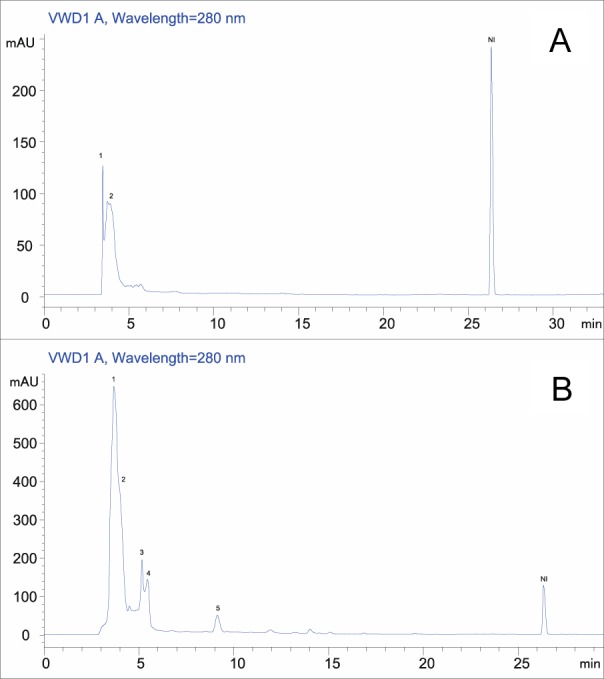
Chromatographic profiles of *Rhus tripartitum* root bark water (A) and methanol (B) extract acquired at 280 nm

In order to explain the strong antioxidant activities of *Rhus tripartitum* extracts, a detailed polyphenol investigation using HPLC was carried out in the root water and methanol fraction since it exhibited the best antioxidant activities. Results showed that the major identified compounds were catechol and kaempferol in both root water (Figure 1A(Fig. 1)) (5.77 and 3.93 mg/g DW) and methanol (Figure 1B(Fig. 1)) fractions (36.49 and 33.96 mg/g DW) and followed by gallic acid, 3.4-dihydroxybenzoic acid and daidzein (12.27, 10.56 and 5.02 mg/g DW respectively) in a smaller amount (Table 4(Tab. 4)). The contribution of these minor phenolic compounds to antioxidant functions could not be thus neglected (Kim et al., 2006[15]). Several investigations on flavonoid derivative revealed a broad variety of anticancer, antibacterial, anti-inflammatory, anti-allergic and antiviral activities (Di Carlo et al., 1999[3]; Montoro et al., 2005[27]). Besides, kaempferol, rutin, morin, and other flavonols as antioxidant agents, displayed valuable activities such as antiallergic, anti-inflammatory, antiviral and anticancer abilities (Saxena et al., 2012[34]). They have also been suggested to play a protective role in liver diseases and cardiovascular diseases (Rice-Evans et al., 1996[32]). Two phenolic compounds such as daidzein and genistein have established to be effective substances to substitute estrogen deficiency in menopause situation. Moreover, soya-derived therapeutic preparations contain these bioactive molecules (Dixon and Ferreira, 2002[4]).

## Conclusion

In summary, this study shows that *Rhus tripartitum *root extracts possess antioxidant and anti-inflammatory activities and were found to be active against lung and colon carcinoma cell lines. Besides, root polar fractions (water and methanol) were found to be rich in phenolic compounds explaining the important biological activities of *Rhus tripartitum*. Moreover, these activities were clearly affected by the nature of extracting solvent and more precisely their increasing polarity. These results enhance the therapeutic virtues of the edible* Rhus tripartitum* roots used in traditional medicines in Tunisia as a source of antioxidant and anti-inflammatory biocompounds with varying polarity, and suggests its use as source of natural chemopreventive agents.

## Acknowledgements

This work was supported by the Tunisian Ministry of Higher Education and Scientific Research and Carthage University. A special thanks to Dr Vakhtang Mshvildadze, Catherine Dussault and Karl Lalancette for their useful assistance in experimentation.

## Conflict of interest

The authors declare that they have no conflict of interest.

## Figures and Tables

**Table 1:**
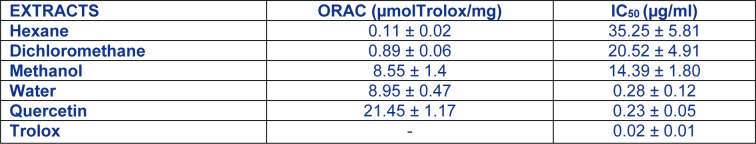
Oxygen radical absorbance capacity (ORAC) values and antioxidant cell assay expressed as IC_50_ of four extracts from *Rhus tripartitum* roots and standards. Each value represents the mean ± SD of three determinations.

**Table 2:**
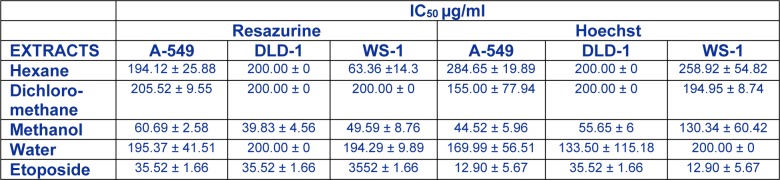
Cytotoxic activity of several extracts from *Rhus tripartitum* against two tumour (A-549, DLD-1) and one healthy (WS1) cell lines.

**Table 3:**
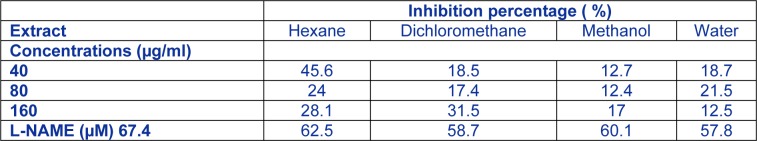
Effect of hexane, methanol, water and dichloromethane extracts from *Rhus tripartitum* on NO overproduction in LPS-stimulated RAW 264.7 macrophages

**Table 4:**
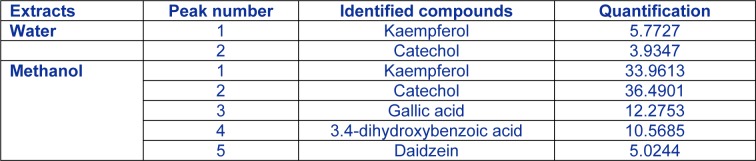
Identified phenolic compounds in *Rhus tripartitum* root bark water and methanol extract. Concentrations are given in milligrams per gram of dry weight (mg. g^−1^ DW).
